# Adverse childhood experiences and adulthood physical performance: the *Brazilian Longitudinal Study of Adult Health* (ELSA-Brasil)

**DOI:** 10.1590/0102-311XEN039125

**Published:** 2026-02-23

**Authors:** Rodrigo Pinto, Sandhi Maria Barreto, Amanda Viana Machado, Ana Luísa Patrão, Luana Giatti, Rosane Harter Griep, Rosa Weiss Telles, Lidyane do Valle Camelo

**Affiliations:** 1 Programa de Pós-graduação em Saúde Pública, Universidade Federal de Minas Gerais, Belo Horizonte, Brasil.; 2 Universidade do Porto, Porto, Portugal.; 3 Fundação Oswaldo Cruz, Rio de Janeiro, Brasil.

**Keywords:** Adverse Childhood Experiences, Child Labor, Physical Performance, Experiências Adversas da Infância, Trabalho Infantil, Desempenho Físico, Experiencias Adversas de la Infancia, Trabajo Infantil, Desempeño Físico

## Abstract

This study aimed to investigate associations between adverse childhood experiences (ACEs) and adulthood physical performance within a Brazilian population using a cross-sectional analysis. A total of 10,896 civil servants, aged 41 to 84 years, from the third wave of the *Brazilian Longitudinal Study of Adult Health*, were included in this research. ACEs were identified retrospectively using child labor and five household dysfunction indicators related to the first 14 years of life, analyzed individually and as a cumulative score. Physical performance was assessed using *Short Physical Performance Battery*, whose score was categorized as poor (≤ 7) or good (≥ 8) physical performance. Binary logistic regression models were employed. Participants had a mean age of 59 years (SD = 8.71), with 42% reporting exposure to at least 1 ACE and 28% having poor physical performance. After adjustments, exposure to one (OR = 1.18; 95%CI: 1.07; 1.32), two (OR = 1.19; 95%CI: 1.02; 1.39), and three or more ACEs types (OR = 1.36; 95%CI: 1.05; 1.75) increased poor physical performance chances in adulthood. Those who experienced household members conviction/incarceration (OR = 1.43; 95%CI: 1.02; 1.99), parental death (OR = 1.22; 95%CI: 1.05; 1.41) or separation/divorce (OR = 1.17; 95%CI: 1.01; 1.36) during childhood also had higher poor physical performance chances in adulthood. ACEs are associated with increased poor physical performance chances in adulthood. These findings underscore the importance of public policies aimed at reducing ACEs to alleviate the burden of diminished physical performance in adulthood.

## Abstract (pt)

O objetivo foi investigar associações entre experiências adversas na infância (EAIs) e o desempenho físico na vida adulta em uma população brasileira. Uma análise transversal foi realizada com 10.896 servidores públicos, com idades entre 41 e 84 anos, da terceira onda do *Estudo Longitudinal de Saúde do Adulto*. As EAIs foram identificadas retrospectivamente utilizando trabalho infantil e cinco indicadores de disfunção domiciliar antes dos 14 anos de idade, analisados individualmente e por meio do escore cumulativo. O desempenho físico foi avaliado usando a *Short Physical Performance Battery* [Bateria Curta de Desempenho Físico], cujo escore foi categorizado como desempenho físico baixo (≤ 7) ou alto (≥ 8). Modelos de regressão logística binária foram empregados. Os participantes tinham uma idade média de 59 anos (DP = 8,71). Um total de 42% relatou exposição a pelo menos uma EAI e 28% tiveram baixo desempenho físico. Após os ajustes, a exposição a um (OR = 1,18; IC95%: 1,07; 1,32), dois (OR = 1,19; IC95%: 1,02; 1,39) e três ou mais tipos de EAI (OR = 1,36; IC95%: 1,05; 1,75) aumentou as chances de baixo desempenho físico na vida adulta. Aqueles que vivenciaram a condenação/encarceramento de membros da família (OR = 1,43; IC95%: 1,02; 1,99), a morte de mãe e/ou pai (OR = 1,22; IC95%: 1,05; 1,41) e a separação/divórcio dos pais (OR = 1,17; IC95%: 1,01; 1,36) durante a infância também apresentaram chances maiores de baixo desempenho físico na vida adulta. EAIs associam-se ao aumento das chances de baixo desempenho físico na vida adulta. Essas descobertas ressaltam a importância de políticas públicas voltadas à redução das EAIs para minimizar a ocorrência do baixo desempenho físico na vida adulta.

## Abstract (es)

El objetivo era investigar las asociaciones entre las experiencias adversas en la infancia (EAIs) y el desempeño físico en la edad adulta en una población brasileña. Se realizó un análisis transversal con 10.896 funcionarios públicos, de entre 41 y 84 años, de la tercera fase del *Estudio Longitudinal de Salud del Adulto*, que fueron incluidos en esta investigación. Las EAIs se identificaron retrospectivamente utilizando indicadores de trabajo infantil y cinco indicadores de disfunción familiar antes de los 14 años, analizados individualmente y como puntuación acumulativa. El desempeño físico se evaluó utilizando la *Short Physical Performance Battery* [Batería de Desempeño Físico Corto], cuya puntuación se clasificó como desempeño físico deficiente (≤ 7) o bueno (≥ 8). Se emplearon modelos de regresión logística binaria. Los participantes tenían una edad media de 59 años (DE = 8,71), el 42% declaró haber estado expuesto al menos a una EAI y el 28% presentaba un desempeño físico deficiente. Tras los ajustes, la exposición a un (OR = 1,18; IC95%: 1,07; 1,32), dos (OR = 1,19; IC95%: 1,02; 1,39) y tres o más tipos de EAIs (OR = 1,36; IC95%: 1,05; 1,75) aumentaba las posibilidades de un desempeño físico deficiente en la edad adulta. Quienes vivieron la condena/encarcelamiento de miembros de la familia (OR = 1,43; IC95%: 1,02; 1,99), sufrir la muerte de los padres (OR = 1,22; IC95%: 1,05; 1,41) o la separación/divorcio (OR = 1,17; IC95%: 1,01; 1,36) durante la infancia también aumentaba las posibilidades de un desempeño físico deficiente en la edad adulta. Las EAIs se asocian con un mayor riesgo de bajo desempeño físico en la edad adulta. Estos hallazgos subrayan la importancia de las políticas públicas destinadas a reducir las EAIs para aliviar la carga que supone el bajo desempeño físico en la edad adulta.

## Introduction

Physical performance encompasses a range of bodily skills that are crucial for daily activities, including locomotor functions, muscular strength, balance, dexterity, and resistance ^1^ ^,^ ^2^ ^,^ ^3^. In epidemiological studies, it is typically assessed via standardized tests measuring gait speed, balance, chair stand, and handgrip strength ^1^ ^,^ ^2^ ^,^ ^3^ ^,^ ^4^. Poor physical performance in adulthood correlates with adverse outcomes such as falls, hospitalizations, increased healthcare costs, diminished quality of life, and higher mortality ^1^ ^,^ ^3^ ^,^ ^4^ ^,^ ^5^ ^,^ ^6^ ^,^ ^7^ ^,^ ^8^ ^,^ ^9^ ^,^ ^10^ ^,^ ^11^ ^,^ ^12^.

The aging process naturally diminishes physical performance, which can be exacerbated by unfavorable socioenvironmental factors experienced early in life, including adverse childhood experiences (ACEs) ^5^ ^,^ ^7^ ^,^ ^13^ ^,^ ^14^. ACEs encompass traumatic events occurred within the household environment, such as exposure to parental substance abuse or incarceration ^15^. Similar to poor physical performance, ACEs show a remarkable impact on health outcomes in adulthood ^15^ ^,^ ^16^ ^,^ ^17^ ^,^ ^18^ ^,^ ^19^, mediated via mechanisms such as stress-induced neurobiological changes and adverse social and behavioral trajectories ^20^.

However, there are few studies linking ACEs to physical performance in adulthood and they often overlook potential confounding variables, such as race/skin color, or are constrained with small sample sizes, hindering the identification of precise associations ^5^ ^,^ ^7^ ^,^ ^14^. Moreover, most studies are limited to assessing exposure to ACEs via cumulative scores, without accounting for the potential specific effects of each experience on adulthood physical performance ^5^ ^,^ ^7^ ^,^ ^14^.

ACEs are particularly relevant in low- and middle-income countries such as Brazil, where a sizable portion of the population lives in poverty and may be highly exposed to ACEs ^21^. Additionally, identifying modifiable factors that influence healthy aging is imperative in Brazil, given its growing aging population ^22^ ^,^ ^23^. The diverse population of Brazil also contends with structural racism ^24^, which may influence ACEs exposure and physical performance in adulthood via several mechanisms, such as poverty, unemployment, and violence in communities.

This study aims to investigate associations between ACEs indicators - both individually and cumulatively - with physical performance in Brazilian adults. The hypothesis posits that higher ACEs exposure is associated with poorer physical performance in adulthood.

## Material and methods

### Study design and population

**Figure 1:**
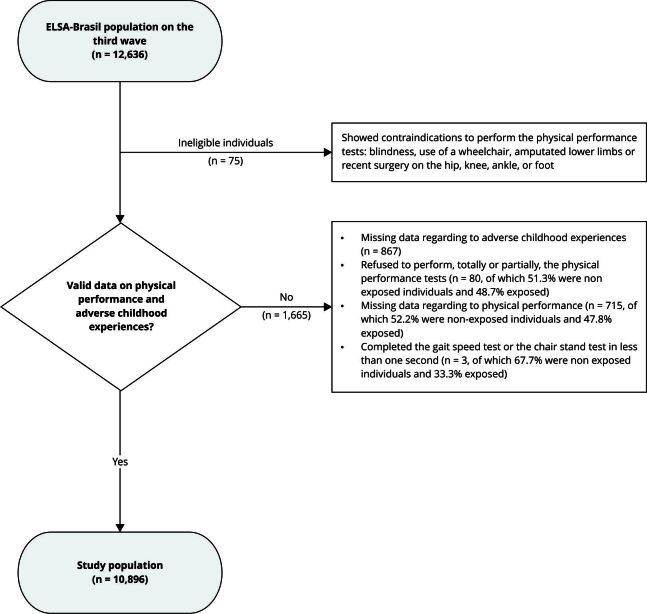
Study participation flowchart. *Brazilian Longitudinal Study of Adult Health* (ELSA-Brasil), 2017-2019.

This study adopts a cross-sectional design with a longitudinal perspective, retrospectively assessing exposure to ACEs. Data were sourced from the third wave (2017-2019) of the *Brazilian Longitudinal Study of Adult Health* (ELSA-Brasil, acronym in Portuguese), for which additional information is available elsewhere ^25^ ^,^ ^26^. Of the 12,636 participants in the ELSA-Brasil third wave, we included 10,896 individuals with valid data on adulthood physical performance and ACEs (Figure 1). All tests and questionnaires were administered by trained and certified research assistants to minimize systematic errors and ensure data quality.

### Response variable

Physical performance was evaluated using the *Short Physical Performance Battery* (SPPB) ^4^ ^,^ ^27^, encompassing gait speed, balance, and chair stand tests, measured in seconds.

(i) Gait speed test. Participants walked for four meters at their usual speed; each participant performed the test twice. The test performed in the shortest time was used for analysis.

(ii) Balance test. Conducted in three positions with progressive difficulty levels: feet side-by-side, semi-tandem, and tandem. The test ended when the participant became unbalanced or after remaining in each position for the maximum time (10 seconds). Participants were only allowed to move to the next position if they remained in the previous position for the maximum time.

(iii) Chair stand test. Participants were asked to stand up from a chair as quickly as possible five times in a row. Each participant completed the test twice. The test performed in the shortest time was used for analysis.

All the tests were scored according to the method proposed by Guralnik et al. ^4^, in 1994. Specifically, scores for each test ranged from 0 to 4 - the higher the score, the better the physical performance. To categorize individuals into these scores, we utilized the distribution of the times taken to complete gait speed and chair stand tests, separately, dividing them into quartiles. The first quartile (representing the fastest participants) scored 4, the second 3, the third 2, and the fourth (the slowest) scored 1. Those unable to complete each test received a score of 0. Scoring was not specific to sex or age, as performances overlapped in the levels of these characteristics (Supplementary Material - Figures S1 and S2; https://cadernos.ensp.fiocruz.br/static//arquivo/suppl-e00039125_8214.pdf). In the balance test, participants received a score of 0 if they were unable to maintain the feet side-by-side position for 10 seconds; 1 if they could hold the side-by-side position for 10 seconds but not the semi-tandem position for 10 seconds; 2 if they could hold the semi-tandem position for 10 seconds but not the tandem position for more than 2 seconds; 3 if they could hold the tandem position for between 3 and 9 seconds; and finally, they received a score of 4 if they could hold the tandem position for 10 seconds.

The final SPPB score consisted of the simple sum of the scores for each test, ranging from 0 to 12. Subsequently, this score was dichotomized, according to recommendation from other studies ^14^ ^,^ ^28^ ^,^ ^29^, into poor (7 points or fewer) or good (8 points or more) physical performance.

### Explanatory variable of interest

ACEs assessment comprised five household dysfunction indicators and one related to child labor before 14 years. Household dysfunction indicators were derived from the *Adverse Childhood Experiences International Questionnaire* ^30^, encompassing parental/guardian death or separation/divorce, in addition to substance abuse, mental illness, and incarceration of household members. Individuals were classified as exposed to child labor if they engaged in work activities before 14 years old, but only when these activities interfered with schooling or led to school dropout. We defined child labor in these terms to improve specificity, once an elevated proportion of our population (24.3%) reported having worked during their first 14 years of life. Highly sensitive experiences, such as physical, sexual, and emotional abuse, were not assessed in ELSA-Brasil. This decision was to prevent participants’ discomfort, non-responses, and withdrawals. Responses were dichotomized (yes = 1, no = 0) and summed for a cumulative score ranging from 0 to 6. Categorical classification of this score included no exposure, exposure to one, exposure to two and exposure to three or more types of ACEs.

### Other covariates

Potential confounders, according to the theoretical model shown in Supplementary Material (Figure S3; https://cadernos.ensp.fiocruz.br/static//arquivo/suppl-e00039125_8214.pdf), encompassed age (continuous), sex (male/female), race/skin color (white; mixed-race; black), maternal education and paternal education (high school or higher; elementary education; primary education; had not attended school). In this study, maternal and paternal education were used as indicators of childhood socioeconomic position. White individuals (n = 6,000) were analyzed together with individuals with Asian origins (n = 296) due to the limited number of the latter, and because they had similar prevalence of ACEs and physical performance characteristics in this study. Similarly, due to limited number (n = 101) and the historical social vulnerability and exclusion of Brazilian Indigenous people, this group was analyzed together with black individuals (n = 1,534), which also have a history of social disadvantages and marginalization, resulting from structural racism and historical disrespect produced by slavery.

### Statistical analysis

Distributions of all variables were described using proportions. Tetrachoric correlation coefficients were calculated to explore the relationships among the ACEs indicators. Using complete-case analysis, logistic regression models estimated the magnitude of associations between the cumulative score and each ACEs indicator with physical performance in adulthood, via odds ratio (OR) and 95% confidence intervals (95%CI). Sequential model adjustments incorporated age and sex (model 1), race/skin color (model 2), and maternal and paternal education (model 3). Multiplicative interaction terms between sex and ACE indicators were included in the final models. No statistically significant interactions were found; therefore, models were not stratified by sex. Statistical significance was set at 5%. Analysis employed Stata 14.00 software (https://www.stata.com).

### Declaration of generative artifical intelligence (AI) and AI-assisted technologies in the writing process

During the preparation of this work, the authors used ChatGPT (OpenAI; https://chatgpt.com/) to improve readability and language. After using this tool/service, the authors reviewed and edited the content as needed and take full responsibility for the content of the publication.

### Statements of ethical approval

The study procedures were performed in compliance with relevant laws and institutional guidelines and have been approved by the ethics committees of all the involved institutions (Federal University of Bahia - approval n. 027/06; Federal University of Espírito Santo - approval n. 041/06; Federal University of Minas Gerais - approval n. 186/06; Oswaldo Cruz Foundation - approval n. 343/06; Federal University of Rio Grande do Sul - approval n. 194/06; University of São Paulo - approval n. 669/06). Informed consent was obtained from all the participants, and the privacy rights of human subjects were respected.

## Results

Of the 12,636 participants on the third wave of ELSA-Brasil, 12,561 were eligible for this investigation (Figure 1). After excluding individuals who declined to participate in the SPPB assessment, as well as those with missing data regarding ACEs questionnaire and physical performance tests, our investigation analyzed 10,896 individuals (Figure 1). The mean (standard deviation - SD) age of this analytical population was 59.2 (SD = 8.7) years, ranging from 41 to 84 years, with 55.1% being female. Table 1 shows additional sociodemographic descriptions. About 70% of the participants were aged within the range of 51 to 70 years old, and most of them identified themselves as white (Table 1). Notably, a significant proportion of individuals had mothers with low levels of education: 43% of these mothers had completed only primary education (up to four years of study), while 12.8% had never attended school (Table 1).

**Table 1:** Distribution of sociodemographic variables, childhood socioeconomic position and exposure to adverse childhood experiences (ACEs) according to physical performance in adulthood. *Brazilian Longitudinal Study of Adult Health* (ELSA-Brasil), 2017-2019 (n = 10,896).

Variables	Total	Physical performance	
		Poor (≤ 7 points)	Good (≥ 8 points)
	n (%)	n = 3,048 (%)	n = 7,848 (%)
Sociodemographic parameters			
Sex			
Male	4,897 (44.9)	1,103 (22.5)	3,794 (77.5)
Female	5,999 (55.1)	1,945 (32.4)	4,054 (67.6)
Age group (years)			
41-50	1,771 (16.3)	310 (17.5)	1,461 (82.5)
51-60	4,595 (42.1)	1,055 (23.0)	3,540 (77.0)
61-70	3,299 (30.3)	1,076 (32.6)	2,223 (67.4)
71 or more	1,231 (11.3)	607 (49.3)	624 (50.7)
Race/Skin color *			
White **	6,296 (58.4)	1,591 (25.3)	4,705 (74.7)
Brown	2,850 (26.4)	808 (28.3)	2,042 (71.7)
Black ***	1,635 (15.2)	609 (37.2)	1,026 (62.8)
Childhood socioeconomic position			
Maternal education *			
High school or higher	2,686 (25.0)	569 (21.2)	2,117 (78.8)
Elementary education	2,066 (19.2)	577 (27.9)	1,489 (72.1)
Primary education	4,621 (43.0)	1,281 (27.7)	3,340 (72.3)
Had not attended school	1,371 (12.8)	553 (40.3)	818 (59.7)
Paternal education *			
High school or higher	3,409 (31.3)	796 (23.3)	2,613 (76.7)
Elementary education	2,051 (18.8)	551 (26.9)	1,500 (73.1)
Primary education	4,074 (37.4)	1,142 (28.0)	2,932 (72.0)
Had not attended school	881 (4.4)	361 (41.0)	520 (59.0)
ACEs			
Child labor			
No	10,168 (93.3)	2,788 (27.4)	7,380 (72.6)
Yes	728 (6.7)	260 (35.7)	468 (64.3)
Having lived with household members who were problem drinkers, had alcohol disorder or misused street or prescription drugs			
No	8,638 (79.3)	2,396 (27.7)	6,242 (72.3)
Yes	2,258 (20.7)	652 (28.9)	1,606 (71.1)
Having lived with household members who were depressed, mentally ill, or suicidal			
No	9,927 (91.1)	2,777 (27.0)	7,150 (72.0)
Yes	969 (8.9)	271 (27.0)	698 (72.0)
Having lived with household members who were sent to jail or prison			
No	10,714 (98.3)	2,986 (27.9)	7,728 (72.1)
Yes	182 (1.7)	62 (34.1)	120 (65.9)
Parental separation/divorce			
No	9,596 (88.1)	2,650 (27.6)	6,946 (72.4)
Yes	1,300 (11.9)	398 (30.6)	902 (69.4)
Death of parents or guardians			
No	9,717 (89.2)	2,649 (27.3)	7,068 (72.7)
Yes	1,179 (10.8)	399 (33.8)	780 (66.2)

* Race/Skin color has 115 missing individuals; maternal education 152 missing individuals; paternal education 481 missing individuals;

** White individuals (n = 6,000) were analyzed together with individuals with Asian origins (n = 296);

*** Black individuals (n = 1,534) were analyzed together with Brazilian Indigenous individuals (n = 101).

Overall, 42.2% of the population reported exposure to at least one type of ACE, with 28.1% reporting exposure to one type, 10.5% to two types, and 3.6% to three or more types. Exposure to ACEs was more prevalent among females, younger groups, black and brown individuals, and those from poorer socioeconomic position during childhood (Table 2). Tetrachoric correlation coefficients indicated significant weak relationships among ACEs indicators, ranging from 0.070 to 0.370 (Table 3).

**Table 2:** Distribution of sociodemographic variables and childhood socioeconomic position according to exposure to adverse childhood experiences (ACEs). *Brazilian Longitudinal Study of Adult Health* (ELSA-Brasil), 2017-2019 (n = 10,896).

Variables	Total	ACEs	
		Exposed *	Not exposed
	n (%)	n = 4,596 (%)	n = 6,300 (%)
Sociodemographic variables			
Sex			
Male	4,897 (44.9)	1,972 (40.3)	2,925 (59.7)
Female	5,999 (55.1)	2.624 (43,7)	3,375 (56.3)
Age group (years)			
41-50	1,771 (16.3)	830 (46.9)	941 (53.1)
51-60	4,595 (42.1)	2,065 (44.9)	2,530 (55.1)
61-70	3,299 (30.3)	1,313 (39.8)	1,986 (60.2)
71 or more	1,231 (11.3)	388 (31.5)	843 (68.5)
Race/Skin color **			
White ***	6,296 (58.4)	2,240 (35.6)	4,056 (64.4)
Mixed-race	2,850 (26.4)	1,387 (48.7)	1,463 (51.3)
Black ^#^	1,635 (15.2)	929 (56.8)	706 (43.2)
Childhood socioeconomic position			
Maternal education *			
High school or higher	2,686 (25.0)	937 (34.9)	1,749 (65.1)
Elementary education	2,066 (19.2)	829 (40.1)	1,237 (59.9)
Primary education	4,621 (43.0)	1,981 (42.9)	2,640 (57.1)
Had not attended school	1,371 (12.8)	741 (54.1)	630 (45.9)
Paternal education *			
High school or higher	3,409 (31.3)	1,125 (33.0)	2,284 (67.0)
Elementary education	2,051 (18.8)	839 (40.9)	1,212 (59.1)
Primary education	4,074 (37.4)	1,773 (43.5)	2,301 (56.5)
Had not attended school	881 (4.4)	497 (56.4)	384 (43.6)

* Defined as exposure to, at least, one of the following situations: child labor interfering with education or leading to school dropout, parental/guardian death or separation/divorce, substance abuse, mental illness, and incarceration of household members;

** Race/Skin color has 115 missing individuals; maternal education 152 missing individuals; paternal education 481 missing individuals;

*** White individuals (n = 6,000) were analyzed together with individuals with Asian origins (n = 296);

# Black individuals (n = 1,534) were analyzed together with Brazilian Indigenous individuals (n = 101).

**Table 3:** Tetrachoric correlation coefficients for the indicators of adverse childhood experiences.

	Child labor	Having lived with household members who were problem drinkers, had alcohol disorder or misused street or prescription drugs	Having lived with household members who were depressed, mentally ill, or suicidal	Having lived with household members who were sent to jail or prison	Parental separation/divorce	Death of parents or guardians
Child labor	1.000					
Having lived with household members who had drinking and alcohol disorder or misused street or prescription drugs	0.291 *	1.000				
Having lived with household members who were depressed, mentally ill or suicidal	0.115 *	0.331 *	1.000			
Having lived with household members who were sent to jail or prison	0.190 *	0.370 *	0.269 *	1.000		
Parental separation/divorce	0.217 *	0.261 *	0.180 *	0.211 *	1.000	
Death of parents or guardians	0.226 *	0.120 *	0.070 **	0.151 *	0.085 *	1.000

* p-value < 0.01;

** p-value < 0.05.

**Figure 2:**
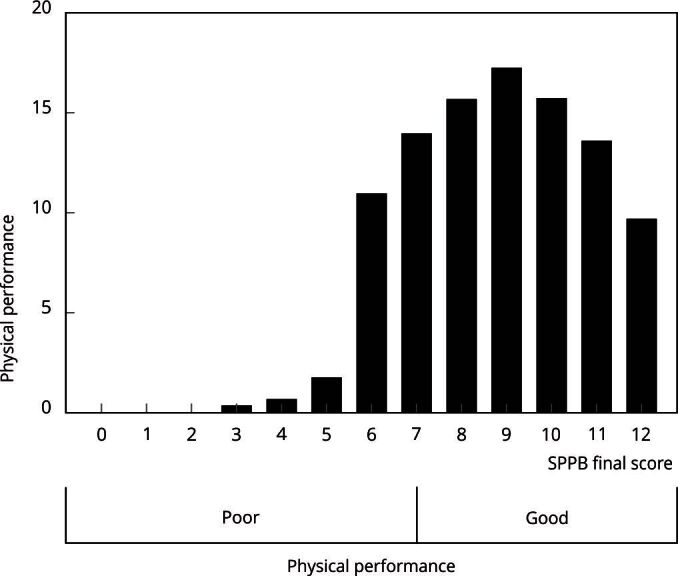
*Short Physical Performance Battery* (SPPB) scores distribution. *Brazilian Longitudinal Study of Adult Health* (ELSA-Brasil), 2017-2019.

Figure 2 illustrates the distribution of the SPPB final score. When scoring the gait speed test, the quartiles cutoff points were 3.31 seconds, 3.69 seconds, and 4.29 seconds; for the chair stand test, they were 7.47 seconds, 8.83 seconds, and 10.50 seconds. A total of 28% of the population showed poor physical performance in adulthood (Figure 2), which was higher among females, older groups, black individuals, participants with parents who had never attended school, and among those exposed to the ACEs indicators (Table 1).

**Figure 3:**
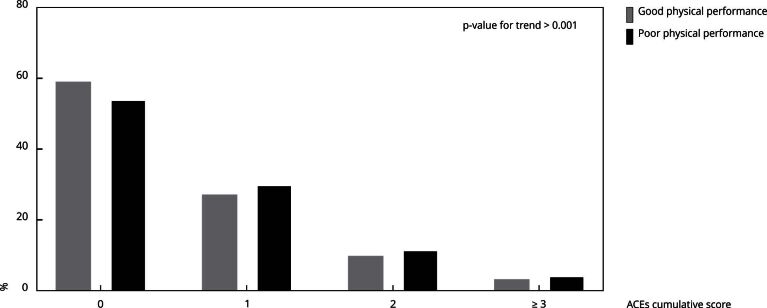
Levels of physical performance according to the adverse childhood experiences (ACEs) cumulative score. *Brazilian Longitudinal Study of Adult Health* (ELSA-Brasil), 2017-2019.

Figure 3 demonstrates an inverse relationship between ACEs cumulative score and physical performance in adulthood: good physical performance was more prevalent among non-exposed individuals, while poor physical performance was more common among those exposed to one, two, and three or more ACEs indicators. This finding suggests a significant trend in the relationship between ACEs cumulative score and physical performance in adulthood (p-value for trend < 0.001). 

Final logistic regression models revealed that the chances of poor physical performance in adulthood increased by 18% for those exposed to one type (OR = 1.18; 95%CI: 1.07; 1.32), 19% for those exposed to two types (OR = 1.19; 95%CI: 1.02; 1.39), and 36% for those exposed to three or more types of ACEs (OR = 1.36; 95%CI: 1.05; 1.75) compared to non-exposed individuals (Table 4). These associations hold a significant upward trend (Wald test for trends p-value < 0.01).

**Table 4:** Associations of the adverse childhood experiences (ACEs) cumulative score and of each indicator of ACEs with poor physical performance: crude model and adjusted models. *Brazilian Longitudinal Study of Adult Health* (ELSA-Brasil), 2017-2019.

Variables	Crude model	Model 1	Model 2	Final model
OR (95%CI)	OR (95%CI)	OR (95%CI)	OR (95%CI)
ACEs cumulative score				
No exposure	Reference	Reference	Reference	Reference
Exposure to one type	1.20 (1.09; 1.32) *	1.30 (1.18; 1.43) *	1.23 (1.11; 1.36) *	1.18 (1.07; 1.32) **
Exposure to two types	1.27 (1.10; 1.45) **	1.40 (1.22; 1.62) *	1.29 (1.11; 1.49) **	1.19 (1.02; 1.39) ***
Exposure to three or more types	1.37 (1.10; 1.70) **	1.66 (1.32; 2.08) *	1.44 (1.15; 1.81) **	1.36 (1.05; 1.75) ***
Child labor				
No	Reference	Reference	Reference	Reference
Yes	1.47 (1.26; 1.72) *	1.55 (1.31; 1.82) *	1.36 (1.15; 1.61) *	1.19 (0.99; 1.43)
Having lived with household members who were problem drinkers, had alcohol disorder or misused street or prescription drugs				
No	Reference	Reference	Reference	Reference
Yes	1.06 (0.95; 1.17)	1.22 (1.09; 1.35) *	1.14 (1.02; 1.27) ***	1.10 (0.98; 1.23)
Having lived with household members who were depressed, mentally ill, or suicidal				
No	Reference	Reference	Reference	Reference
Yes	1.00 (0.86; 1.16)	1.01 (0.87; 1.18)	1.03 (0.89; 1.20)	1.05 (0.90; 1.23)
Having lived with household members who were sent to jail or prison				
No	Reference	Reference	Reference	Reference
Yes	1.34 (0.98; 1.82)	1.45 (1.05; 2.00) ***	1.37 (0.99; 1.89)	1.43 (1.02; 1.99) ***
Parental separation/divorce				
No	Reference	Reference	Reference	Reference
Yes	1.16 (1.02; 1.31) ***	1.35 (1.18; 1.54) *	1.24 (1.08; 1.41) **	1.17 (1.01; 1.36) ***
Death of parents or guardians				
No	Reference	Reference	Reference	Reference
Yes	1.36 (1.20; 1.55) *	1.32 (1.15; 1.51) *	1.23 (1.07; 1.40) **	1.22 (1.05; 1.41) ***

95%CI: 95% confidence interval; OR: odds ratio.

Note: poor physical performance defined by *Short Physical Performance Battery* final score ≤ 7 points. Crude model: without adjustments; model 1: adjusted for age and sex; model 2: adjusted for model 1 and race/skin color; final model: adjusted for model 2, maternal and paternal education.

* p-value < 0.001;

** p-value < 0.01;

*** p-value < 0.05.

Varied ORs were observed when each ACE indicator was analyzed separately in logistic regression models. While the crude model indicated a 47% higher chance of poor physical performance in adulthood for those exposed to child labor compared to non-exposed individuals (OR = 1.47; 95%CI: 1.26; 1.73), this association lost statistical significance in the final model (Table 4). Nevertheless, even after adjusting for all confounders, those who experienced conviction/incarceration of household members, parental separation/divorce and parental/guardian death during childhood had their chances of poor physical performance in adulthood increased by 43% (OR = 1.43; 95%CI: 1.02; 1.99), 17% (OR = 1.17; 95%CI: 1.01; 1.36), and 22% (OR = 1.22; 95%CI: 1.05; 1.41), respectively (Table 4).

## Discussion

This study, situated in a Brazilian context, explored the associations between six different indicators of ACEs, related to the first 14 years of life, with physical performance in adulthood. Our findings revealed that, cumulatively, ACEs were associated with poor physical performance in adulthood, with the strongest association observed in individuals exposed to three or more types of ACEs. These associations held a significant upward trend. Our findings build upon previous studies by showing that, independently of confounders, individuals who had a household member arrested, or experienced parental separation or death during childhood, had poorer physical performance in adulthood.

The observed associations between cumulative exposure to ACEs and poor physical performance in adulthood align with previous research findings. A multinational study among older adults in Brazil, Colombia, Albania, and Canada demonstrated that those most exposed to ACEs had a 236% higher chance of poor physical performance in adulthood ^14^. However, this result was not adjusted for race/skin color, a significant confounding factor in our analysis. Additionally, it includes physical and emotional abuse as ACEs indicators, which were not investigated in our study. Another research, situated in United Kingdom, showed that the greater the exposure to ACEs, the poorer the mean physical performance score in adulthood ^7^. Nonetheless, unlike the results of this study, the association between ACEs and physical performance in adulthood was not independent of childhood socioeconomic position, measured by paternal occupation, in a cohort of English women ^5^. Note that, although most of these findings are consistent with ours, they come from studies that were conducted in older populations than the ELSA-Brasil cohort. In any case, our results were adjusted for age and showed that, even in a younger population, ACEs could compromise physical performance.

We found no statistically significant interactions between sex and ACEs in our study, and physical performance outcomes were overlapped by sex (Supplementary Material - Figures S1 and S2; https://cadernos.ensp.fiocruz.br/static//arquivo/suppl-e00039125_8214.pdf). Therefore, our results were not stratified by sex and reflect mean associations applicable to both men and women. Similar findings have been reported in other studies ^7^ ^,^ ^14^.

Using a cumulative ACEs exposure score is advantageous because it increases the statistical power of the analysis ^5^. It also accounts for the co-occurrence of different forms of adversity, which may jointly lead to poorer physical performance in adulthood ^5^. However, the use of such scores assumes that all types of ACEs have a similar contribution to diminish physical performance in adulthood, which may not be true. Thus, it is crucial to examine the associations between each ACE indicator and adulthood physical performance individually.

Our findings suggest that individuals who had a household member that was arrested had the highest chances for poor physical performance in adulthood. This could be attributed to the significant stigma and disruptions in social identity and household arrangement caused by such experience ^31^. Moreover, parental/guardian death and separation were associated with poor physical performance in adulthood. Similarly, a study from ELSA-Brasil found that different ACEs had specific patterns of association with depression in adulthood ^32^. Parental separation, for instance, was associated with a 55% increase in the chances of adulthood depression ^32^. Although parental separation is now common, note that the participants of this study were children between the 1950s and 1970s, when parental separation was a great social stigma in Brazil ^33^. This prevailing view regarding divorce/separation then is likely to have influenced the long-term impacts of this experience in our cohort’s adulthood physical performance.

We chose not to mutually adjust the ACEs indicators in multivariate models due to their interrelated nature and other methodological considerations. A fundamental assumption in causal inference is temporal sequencing: when adjusting the association between two variables (X and Y) for a third variable (Z), Z must precede both X and Y. If this assumption is violated, adjustment for Z may introduce spurious associations or obscure true ones. In our study, establishing a consistent temporal order among certain ACEs is particularly challenging. As such, attempting to isolate independent effects of each ACE via mutual adjustment may lead to imprecise interpretations. Nevertheless, note that tetrachoric correlation showed weak relationships among the indicators of ACEs in the context of our research (Table 3). Then, it is possible that mutually adjusting them would not substantially change our results.

Different mechanisms may explain the relationships between ACEs and physical performance in adulthood ^20^. ACEs can indirectly affect physical performance in adulthood by triggering social disadvantages throughout life, such as poor education, poor jobs, and a higher likelihood of engaging in unhealthy behaviors ^20^. Additionally, exposures to ACEs and social disadvantages are important sources of suffering and can directly trigger short- and long-term biological stress responses, such as changes in the functioning of the hypothalamic-pituitary-adrenal axis, deregulated secretion of hormones and neurotransmitters (e.g. cortisol and catecholamines), and amplification of the inflammatory activity ^20^. Chronic inflammation, in its turn, has a catabolic effect and is central to numerous pathophysiological mechanisms, such as bone loss and sarcopenia ^20^ ^,^ ^34^, which play an important role in physical performance.

Despite robustness, our study has limitations. There is a possibility of residual confounding for the variables indicating childhood socioeconomic position. In addition to mother and father’s education, other variables have been used, such as parents’ income and property ownership. The potential for survival bias must be acknowledged, as participants with poorer physical performance and a higher prevalence of ACEs may have passed away before data collection for this study. Additionally, the healthy worker effect should be considered, once our cohort consists of employed personnel from Brazilian research and high education institutes. Retrospective measurement of socially sensitive household-related issues, such as ACEs, is susceptible to desirability bias, memory limitations, and underreporting ^35^, even when questionaries are administered by trained research assistants. However, this does not imply that such retrospective assessments are invalid. A study has shown that measures of ACEs collected in childhood and in adulthood demonstrated reasonable concordance, supporting the validity of retrospective reports ^35^. The combined effect of these limitations may have resulted in an underestimation of the associations found in our study compared to those expected in the general population. Nonetheless, upon recognizing these limitations, the associations between ACEs and adulthood physical performance observed in our study are evidence applicable to the whole population. Analytical studies focusing on specific subgroups of the general population, such as ELSA-Brasil, can enhance scientific inference by prioritizing internal validity over representativeness, enabling an improved assessment of causal relationship ^36^ ^,^ ^37^ ^,^ ^38^.

Regardless of these limitations, our study provides significant insights. We have strengthened the validity of our findings by using the SPPB, a well-established tool for assessing physical performance ^39^, and adjusting associations for race/skin color, an important confounding factor. Additionally, our analysis included consideration of specific ACEs indicators, rather than solely a cumulative score of exposure, thereby advancing our understanding of the nuanced effects of individual ACEs on physical performance in adulthood.

If the associations found in this study are causal, preventing ACEs or giving adequate psychosocial support to those exposed to them may help reduce the burden of poor physical performance later in life and its associated repercussions, such as diminished ability to perform daily activities, increased risk of falls, hospitalizations, and reduced quality of life. Evidence from the literature indicates that multisectoral and multidisciplinary interventions, some of which are feasible within the Brazilian Unified National Health System (SUS, acronym in Portuguese), can contribute to addressing ACEs ^40^ ^,^ ^41^. For instance, screening for domestic violence during prenatal care appointments or home visits by healthcare professionals has been associated with the prevention of ACEs in countries such as England ^40^ ^,^ ^41^. The English experience has also led to the development of screening tools, games, and therapeutic strategies specifically designed for the primary, secondary, or tertiary prevention of ACEs ^40^ ^,^ ^41^. These resources may be adapted for use within healthcare services, social assistance programs, and schools in Brazil. Although this study did not aim to evaluate the effectiveness of such interventions, the authors believe they may be promising in preventing ACEs and/or mitigating their short and long-term health consequences. Moreover, the creation of dedicated commissions within public administration to coordinate ACE-related strategies, an approach adopted in England, has been identified as a promising governance mechanism ^40^ ^,^ ^41^. In the Brazilian context, incorporating such coordination into existing intersectoral structures may help to align and strengthen efforts across health, education, and social protection systems. Nonetheless, the impact of these actions is constrained without broader improvements in living and working conditions. Given that ACEs are partly shaped by structural determinants such as poverty, unemployment, and community violence, effective prevention also requires tackling these root causes via policies aimed at reducing socioeconomic and racial inequalities and expanding social protection. Strengthening the role of SUS in promoting early-life well-being, by actions like early screening, family-centered care, and intersectoral collaboration, is essential for advancing a more comprehensive approach to the prevention of ACEs in Brazil.

## Data Availability

The research data are available upon request to the corresponding author.
