# Supplementary material for: Adverse childhood experiences and adulthood physical performance: the Brazilian Longitudinal Study of Adult Health (ELSA-Brasil)
Source: Cad Saude Publica. 2026 Feb 23;42:e00039125. doi: 10.1590/0102-311XEN039125 (PMC12928553; doi:10.1590/0102-311XEN039125)

SUPPLEMENTARY MATERIAL

Figure S1 Histograms of performances in the gait speed and chair stand tests.

a) Gait speed test

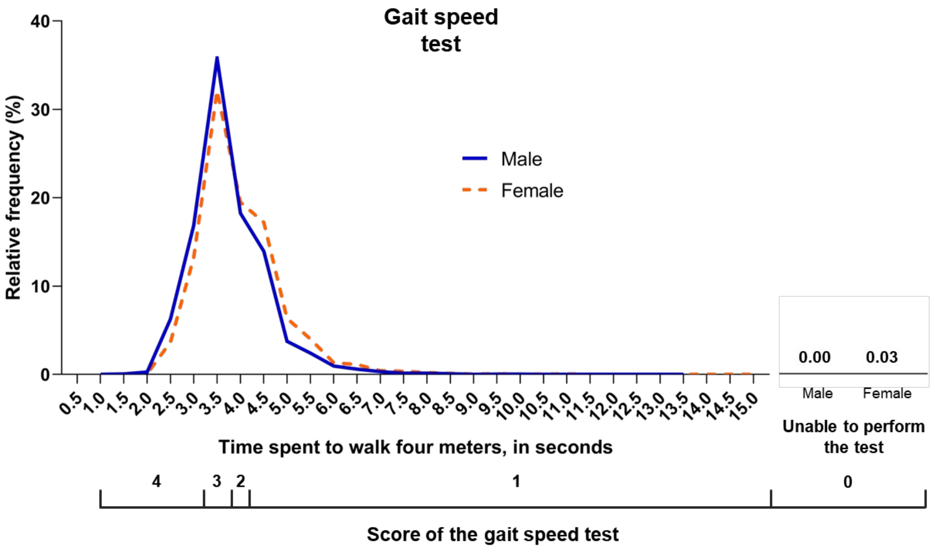

b) Chair stand test

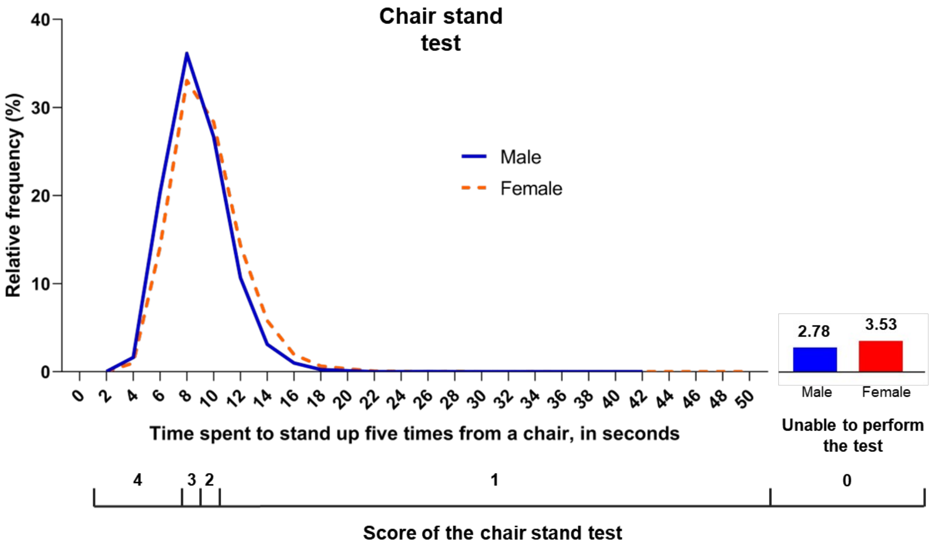

Figure S2 Quartiles of the times of the gait speed and chair stand tests, stratified by sex and age group.

a) Gait speed test

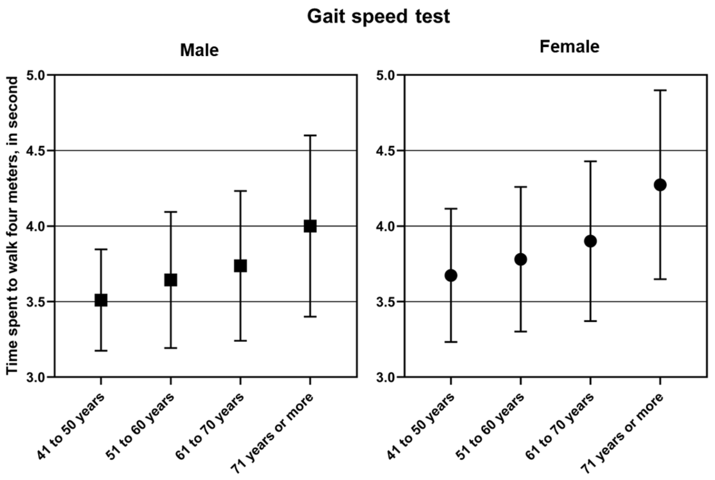

b) Chair stand test

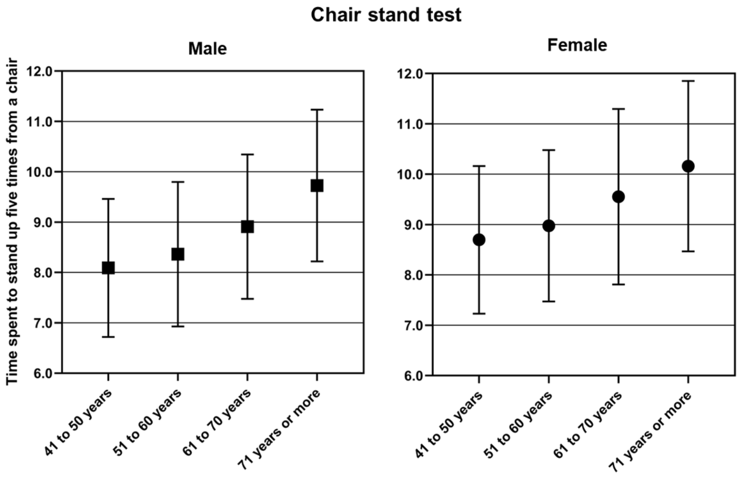

Figure S3 Theoretical model representing the relationships among adverse childhood experiences, physical capability in adulthood, confounding factors and mediating variables.

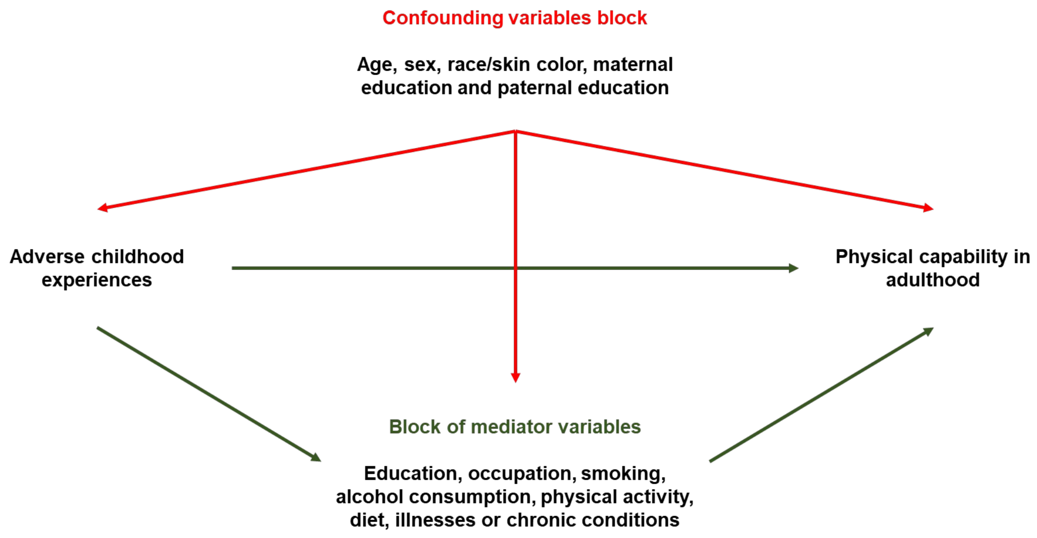

Supplement: Supplementary Material [file 1678-4464-csp-42-EN039125-s.pdf]
